# Evaluation of Ferroptosis as a Biomarker to Predict Treatment Outcomes of Cancer Immunotherapy

**DOI:** 10.1158/2767-9764.CRC-25-0268

**Published:** 2025-08-06

**Authors:** Zhijun Zhou, Yang Cai, Hao Yuan, Qun Chen, Sophia W. Zhao, Jingxuan Yang, Mingyang Liu, Alex X. Arreola, Yu Ren, Chao Xu, Lacey R. McNally, Michael S. Bronze, Courtney W. Houchen, Kuirong Jiang, Wei R. Chen, Yuqing Zhang, Min Li

**Affiliations:** 1 Department of Medicine, The University of Oklahoma Health Sciences Center, Oklahoma City, Oklahoma.; 2 Department of Surgery, The University of Oklahoma Health Sciences Center, Oklahoma City, Oklahoma.; 3 Department of Pathology, The University of Oklahoma Health Sciences Center, Oklahoma City, Oklahoma.; 4 Department of Biostatistics and Epidemiology, Hudson College of Public Health, The University of Oklahoma Health Sciences Center, Oklahoma City, Oklahoma.; 5 Pancreas Center, The Frist Affiliated Hospital of Nanjing Medical University, Nanjing, China.; 6 Stephenson School of Biomedical Engineering, The University of Oklahoma, Norman, Oklahoma.

## Abstract

Immunotherapy has revolutionized the treatment paradigms of several cancer types, yet only a subset of patients derives durable clinical benefit. Ferroptosis is a programmed cell death facilitated by iron-driven overload of lipid peroxidation. We aimed to evaluate ferroptosis activity based on tumor transcriptomic profiles to determine its predictive value for immunotherapy outcomes. We analyzed RNA sequencing data from eight independent cohorts of patients with urothelial, gastric, skin, and lung cancers treated with immune checkpoint inhibitors or adoptive T-cell therapy. A ferroptosis activity model was constructed using downstream gene expression signatures. Associations with overall survival and progression-free survival were assessed. Potential mechanisms were explored by examining immunosenescence, the IFN-γ immune response pathway, and immunogenic cell death. Ferroptosis-high tumors were associated with significantly improved overall survival and progression-free survival across multiple cancer types. Integrating ferroptosis scores with tumor mutation burden, liver metastasis status, and immune microenvironment phenotypes (inflamed, excluded, desert) enhanced patient stratification and predictive accuracy. Mechanistically, ferroptosis enhanced the immune response by promoting immunogenic cell death and attenuating immunosenescence. In conclusion, elevated ferroptosis activity correlates with improved immunotherapy outcomes, potentially through increased tumor immunogenicity and reduced immunosenescence. Ferroptosis-based biomarkers may aid in identifying patients more likely to benefit from immunotherapy.

**Significance:**

Ferroptosis, an iron-dependent cell death process, is linked to improved immunotherapy outcomes. In this real-world study across eight cohorts, ferroptosis-high tumors showed 2 to 3 times longer survival. Mechanistically, ferroptosis enhanced immunogenicity and suppressed immunosenescence, highlighting its potential as a biomarker and therapeutic target to boost immunotherapy efficacy.

## Introduction

The past decade has seen unprecedented progress in cancer immunotherapy, revolutionizing treatment paradigms across several cancer types (1–3). Patients who responded well to immunotherapy often experienced long-term survival and, in some cases, remained tumor-free for years (4). However, only a subset of patients benefited from immunotherapy, underscoring the need to identify novel biomarkers to select patients who could benefit (5). Indeed, several biomarkers, such as tumor mutation burden (TMB), deficient mismatch repair, tertiary lymphoid structures, and *POLE/POLD1* mutations, have been identified as indicators of immunotherapy response (6–15). Although these biomarkers are effective predictors of treatment outcomes, they are not directly targetable to enhance the efficacy of immunotherapy. Moreover, many initially responsive tumors eventually relapse, highlighting the urgent need for rational combination therapies to further improve and sustain treatment efficacy (16–18).

Ferroptosis, a distinct form of cell death characterized by the accumulation of lipid peroxides and iron, has garnered substantial attention in recent years (19–22). Ferroptosis is a key regulator of tumor microenvironment reprogramming (23). In preclinical studies, a feedforward circuit has been observed between tumor ferroptosis and the antitumor activity of T cells (24). Additionally, the administration of ferroptotic tumor cells has been shown to enhance the antitumor effect of immunotherapy (25). Inducing ferroptosis through glutathione peroxidase 4 inhibitors has demonstrated a synergistic effect with immune checkpoint inhibitors (ICI) in breast cancer, an “immune-cold” cancer type characterized by a relatively low response rate to immunotherapy (26). On the other hand, immunotherapy promotes ferroptosis in tumor cells. For instance, the itaconate transporter SLC13A3 promotes tumor immune evasion by inducing the development of ferroptosis-resistant tumor cells and the posttranslational modification of PD-L1 (27, 28). Inhibiting SLC13A3 in tumor cells enhances the efficacy of immunotherapy.

Although several studies have suggested a potential link between ferroptosis and the efficacy of immunotherapy, most of these investigations have been limited to observations in mouse models or in small cohorts of retrospective analyses (29–34). The prognostic significance of ferroptosis in large cohorts of patients with cancer undergoing immunotherapy remains unclear. Moreover, there is currently a lack of robust, ferroptosis-based models for predicting immunotherapy treatment outcomes.

In this study, we constructed a model based on the expression of downstream targets of ferroptosis in the tumor tissue of patients treated with immunotherapy. We found that ferroptosis is associated with treatment outcomes in eight cohorts of patients receiving immunotherapy. Mechanistic studies showed that ferroptosis promotes immune response partially by inducing immunogenic cell death (ICD) and suppressing immunosenescence in tumor tissue.

## Materials and Methods

### Patients and data

This study included eight independent cohorts, the characteristics of which are summarized in Supplementary Table S1. All cohorts consisted of patients who had received immunotherapy, including ICIs or adoptive T-cell therapy. Ethical approval was waived by the institutional ethics committee as all datasets were obtained from publicly available databases, and clinical information had been deidentified.

Cohort 1 is the IMvigor210 cohort, a multicenter clinical trial that enrolled patients with advanced or metastatic urothelial cancers treated with anti–PD-1/PD-L1 inhibitors (35).

Cohort 2 is the Snyder cohort (36), which enrolled patients treated with ipilimumab or tremelimumab. The study aimed to identify genetic factors associated with treatment outcomes in patients treated with anti-CTLA4 inhibitors.

Cohort 3 is the Liu cohort (37), which included patients treated with nivolumab or pembrolizumab. Most tumor samples were collected from metastatic lesions, with the exception of eight samples derived from primary sites. The primary objective was to develop a predictive model for response to ICIs in melanoma.

Cohort 4 is the Van Allen cohort (38), which enrolled patients with metastatic melanoma treated with ipilimumab. The study investigated whether neoantigen load was associated with treatment response and clinical outcomes.

Cohort 5 is the Hugo cohort (39), designed to identify transcriptomic alterations linked to resistance to anti–PD-1 therapy in metastatic melanoma.

Cohort 6 is the Lauss cohort (40), which enrolled patients with metastatic melanoma who received adoptive T-cell therapy. This study aimed to evaluate the predictive value of TMB and neoantigen load for treatment response.

Cohort 7 is the Kim cohort (41), comprising patients with metastatic gastric cancer treated with anti–PD-1 therapy (pembrolizumab). This study aimed to identify biomarkers to indicate response to pembrolizumab.

Cohort 8 consists of patients with non–small cell lung cancer (NSCLC) who were treated with anti–PD-1 therapy (42). This study focused on developing a predictive model for treatment outcomes in patients with NSCLC undergoing immunotherapy.

Cohorts 3 and 7 recorded all-cause mortality, whereas cohort 8 did not report any cause of death. The remaining five cohorts documented cancer-specific mortality. Cohort 6 evaluated the efficacy of adoptive T-cell therapy, whereas the other cohorts evaluated the efficacy of ICIs.

### Outcomes

The primary outcome of this study was overall survival (OS), defined as the time from the first day of immunotherapy administration to the date of death or last follow-up. Secondary outcomes included progression-free survival (PFS) and treatment response. PFS was defined as the interval from the start of treatment to the date of disease relapse.

### Algorithm to evaluate ferroptosis

Ferroptosis downstream target genes were selected based on a previously published study (43). We developed a scoring algorithm based on the expression of these downstream targets in tumor tissue. Specifically, RNA sequencing (RNA-seq) data were collected for 31 genes: *CHAC1*, *DDIT4*, *ASNS*, *TSC22D3*, *DDIT3*, *JDP2*, *SESN2*, *SLC1A4*, *PCK2*, *SLC7A11*, *VLDLR*, *GPT2*, *PSAT1*, *C9ORF150*, *SLC7A5*, *HERPUD1*, *XBP1*, *ATF3*, *SLC3A2*, *CBS*, *ATF4*, *ZNF419*, *KLHL24*, *TRIB3*, *ZNF643*, *ATP6V1G2*, *VEGFA*, *GDF15*, *TUBE1*, *ARRDC3*, and *CEBPG*. As previously described (44), RNA-seq data were log_2_-transformed and subsequently normalized across all samples. A composite ferroptosis score was then calculated for each sample by summing the normalized expression values of all 31 genes. Cutoff values for defining high and low ferroptosis scores were manually determined based on cancer type and treatment context.

### Gene set enrichment analysis

GSEA software (version 4.1.0, RRID:SCR_003199) was used to analyze the enrichment of gene sets between ferroptosis-high and ferroptosis-low tumors. Annotated gene sets of the ferroptosis pathway and immune response pathway were obtained from the Molecular Signatures Database (RRID:SCR_016863). The number of permutations was set to 100. The enrichment statistic was set to weighted.

### Assessment of IFN-γ signaling and immunosenescence-related gene expression

The IFN-γ signature score and expanded immune score were calculated as previously described (45). Briefly, expression data for the relevant genes were obtained, normalized across all samples, and summed to generate a composite score, with higher values reflecting stronger antitumor immune activity. In parallel, we analyzed the expression of a previously defined set of IFN-γ response genes upregulated upon IFN-γ treatment (46). Gene expression levels were extracted and visualized using heatmap analysis in R (version 3.6.3). Elevated expression of these genes was interpreted as indicative of enhanced antitumor immunity. Additionally, curated gene sets associated with the suppression of immunosenescence were retrieved from prior studies (47, 48). Heatmap analyses were conducted in R to assess the expression of these genes, with upregulation suggesting enhanced immune surveillance and reduced immunosenescence in the tumor microenvironment.

### Statistical analysis

Statistical analysis was performed in Prism 10 (GraphPad, RRID:SCR_002798) and R (version 3.6.3, RRID:SCR_001905). The log-rank (Mantel–Cox) test was applied in Kaplan–Meier survival analysis. The cutoff values of the ferroptosis score in each cohort were manually defined unless otherwise specified. The Student *t* test was applied to compare two independent groups. A *P* value less than 0.05 was considered statistically significant.

### Data availability

All datasets are available from the Gene Expression Omnibus (RRID:SCR_005012) database or from original studies, with accession IDs as listed in Supplementary Table S1. All other reasonable requests will be fulfilled by the corresponding authors.

## Results

### Ferroptosis is associated with favorable treatment outcomes in patients receiving immunotherapy

**Figure 1:**
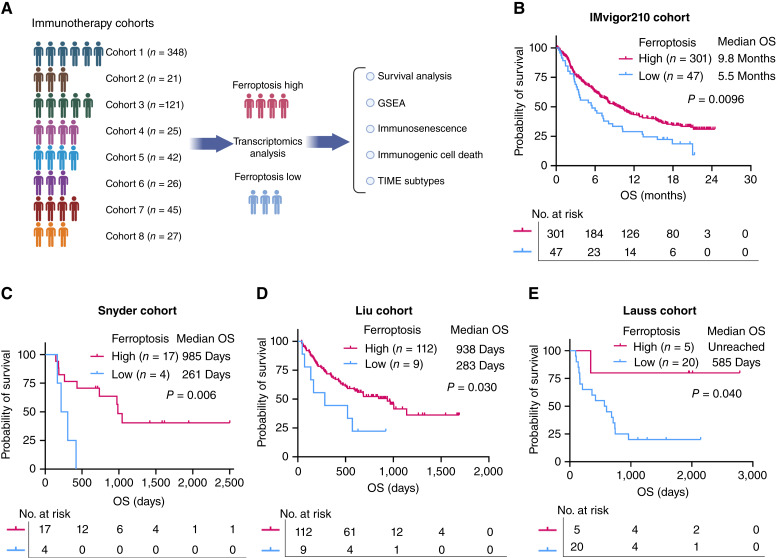
Ferroptosis is associated with favorable treatment outcomes in patients receiving immunotherapy. **A,** The flow chart shows the design of the study. **B,** OS analysis of patients based on ferroptosis levels in tumor tissue in the IMvigor210 cohort (urothelial cancers). **C,** OS analysis of patients based on ferroptosis levels in tumor tissue in the Snyder cohort (melanoma). **D,** OS analysis of patients based on ferroptosis levels in tumor tissue in the Liu cohort (melanoma). **E,** OS analysis of patients based on ferroptosis levels in tumor tissue in the Lauss cohort (melanoma). The log-rank test was applied for the survival analysis. GSEA, gene set enrichment analysis.

A total of 655 patients who received immunotherapy for various cancer types across eight independent cohorts were included in this study. Patients were stratified into ferroptosis-high and ferroptosis-low groups based on tumor gene expression profiles (Fig. 1A). In the IMvigor210 cohort, which includes patients with urothelial cancers, ferroptosis-high tumors were associated with significantly improved OS compared with ferroptosis-low tumors (median OS: 9.8 vs. 5.5 months; *n* = 348; *P* = 0.0096; Fig. 1B). To evaluate whether ferroptosis also serves as a predictive biomarker in other cancer types, we analyzed five additional cohorts of patients with advanced or metastatic melanoma treated with immunotherapy. In the Snyder cohort, patients with ferroptosis-high tumors exhibited markedly better survival outcomes than those with ferroptosis-low tumors (median OS: 985 vs. 261 days; *n* = 21; *P* = 0.006; Fig. 1C). Similarly, in the Liu cohort, ferroptosis-high tumors were associated with longer survival (median OS: 938 vs. 283 days; *n* = 121; *P* = 0.03; Fig. 1D). We further validated these findings in a cohort of patients with melanoma who received adoptive T-cell therapy (Lauss cohort). Patients with ferroptosis-high tumors exhibited significantly improved survival, with median OS unreached after a follow-up of 2,787 days, compared with a median OS of 585 days in the ferroptosis-low group (*n* = 25; *P* = 0.04; Fig. 1E). Additionally, the Van Allen and Hugo cohorts supported the association between ferroptosis and improved immunotherapy responses. Although statistical significance was not reached, likely due to the limited sample sizes, patients with ferroptosis-high tumors consistently demonstrated OS durations 2 to 3 times longer than those with ferroptosis-low tumors (median OS: 853 vs. 293 days, *n* = 42, *P* = 0.156; median OS: unreached vs. 523 days; *n* = 26; *P* = 0.151; Supplementary Fig. S1A and S1B). Furthermore, ferroptosis-high tumors were associated with prolonged PFS following immunotherapy in the Van Allen and Lauss cohorts (median PFS: 211 vs. 77 days, *n* = 42, *P* = 0.145; median PFS: 1,443 vs. 112.5 days; *n* = 25; *P* = 0.098; Supplementary Fig. S2A and S2B). To validate that the ferroptosis score specifically predicts immunotherapy benefit rather than serving as a general prognostic marker, we performed survival analyses in patients with bladder cancer, melanoma, and lung cancer who received standard-of-care therapies without immunotherapy. Our results show that the ferroptosis score is not associated with OS in these cohorts, indicating that it does not hold prognostic significance in the absence of immunotherapy. These findings support our conclusion that the ferroptosis score specifically predicts immunotherapy benefit rather than simply reflecting overall prognosis (Supplementary Fig. S3A–S3C).

Given the recent adoption of immunotherapy as a first-line treatment for advanced gastric cancer (49), we evaluated our ferroptosis-based model in a gastric cancer cohort receiving immunotherapy (41). Patients in the ferroptosis-high group exhibited significantly higher response rates compared with those in the ferroptosis-low group (partial/complete response rate: 45.5% vs. 8.7%; *P* = 0.005; Supplementary Fig. S4A). A similar survival advantage was found in patients with ferroptosis-high gastric cancer treated with chemotherapy, in which the median OS was unreached compared with 74 months in the ferroptosis-low group (*n* = 134; *P* = 0.02; Supplementary Fig. S4B). We also assessed this model in a cohort of patients with NSCLC. Consistent with other cancer types, the ferroptosis-high patients had improved prognosis (median PFS: 257 vs. 52 days; *n* = 27; *P* = 0.235; Supplementary Fig. S5A) though statistical significance was not reached due to the small sample size. Notably, the prognostic value of ferroptosis seemed to be independent of TMB (Supplementary Fig. S5B). Collectively, these findings further support an association between high ferroptosis activity and favorable treatment outcomes across multiple cancer types in the context of immunotherapy.

### Ferroptosis complements liver metastasis and TMB to predict immunotherapy outcomes

**Figure 2:**
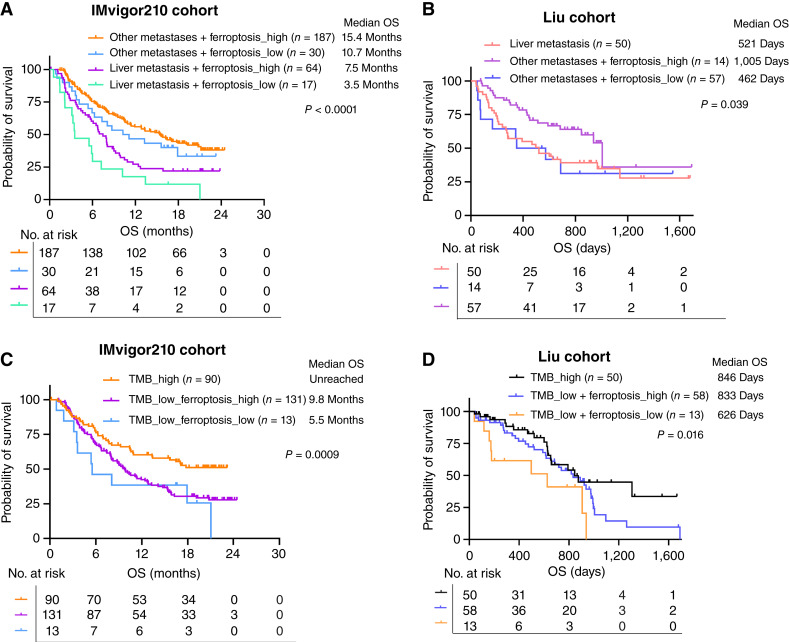
Ferroptosis complements liver metastasis and TMB to predict immunotherapy outcomes. **A,** OS analysis of patients based on the status of liver metastasis and ferroptosis in tumor tissue in the IMvigor210 cohort. **B,** OS analysis of patients based on the status of liver metastasis and ferroptosis in tumor tissue in the Liu cohort. **C,** OS analysis of patients based on TMB and ferroptosis in tumor tissue in the IMvigor210 cohort. **D,** OS analysis of patients based on TMB and ferroptosis in tumor tissue in the Liu cohort. The log-rank test was applied for the survival analysis.

Liver metastasis and TMB are well-established predictors of immunotherapy efficacy (50). We investigated whether ferroptosis could provide additional prognostic value when considered alongside these biomarkers. In the IMvigor210 cohort, patients with liver metastasis had significantly shorter OS compared with those with metastases to other sites (median OS: 7.5/3.5 vs. 15.4/10.7 months; *n* = 298; Fig. 2A). Notably, ferroptosis further stratified outcomes within both subgroups. Among patients with liver metastases, those with ferroptosis-high tumors had improved OS compared with ferroptosis-low counterparts (median OS: 7.5 vs. 3.5 months; *n* = 81; Fig. 2A). Similarly, in patients with other metastatic sites, ferroptosis-high tumors were associated with longer OS (median OS: 15.4 vs. 10.7 months; *n* = 217; Fig. 2A). These findings were validated in the Liu cohort. Consistent with prior observations, those with liver metastases exhibited a worse prognosis compared with those with other metastases (Fig. 2B). Among patients without liver metastases, ferroptosis-high again correlated with superior prognosis (median OS: 1,005 vs. 462 days; *n* = 71; Fig. 2B).

TMB is another established biomarker of immunotherapy response (51). In the IMvigor210 cohort, TMB-high tumors were associated with better outcomes compared with those with TMB-low tumors (median OS: unreached vs. 9.8/5.5 months; *n* = 234; Fig. 2C). To explore the interaction between TMB and ferroptosis, we stratified TMB-low tumors into ferroptosis-low and ferroptosis-high groups. Intriguingly, we found that patients with TMB-low but ferroptosis-high tumors had significantly improved survival (median OS: 9.8 vs. 5.5 months; *n* = 144; Fig. 2C). This result was further validated in the Liu cohort (median OS: 833 vs. 626 days; *n* = 71; Fig. 2D). Importantly, TMB levels are comparable between ferroptosis-high and ferroptosis-low tumors across all cohorts except the Snyder cohort, indicating that ferroptosis enhances immunotherapy responsiveness independent of TMB status (Supplementary Fig. S6A–S6F). Taken together, these findings indicate that ferroptosis complements liver metastasis and TMB to predict immunotherapy outcomes.

### Ferroptosis enhances immune response and suppresses immunosenescence in the tumor microenvironment

**Figure 3:**
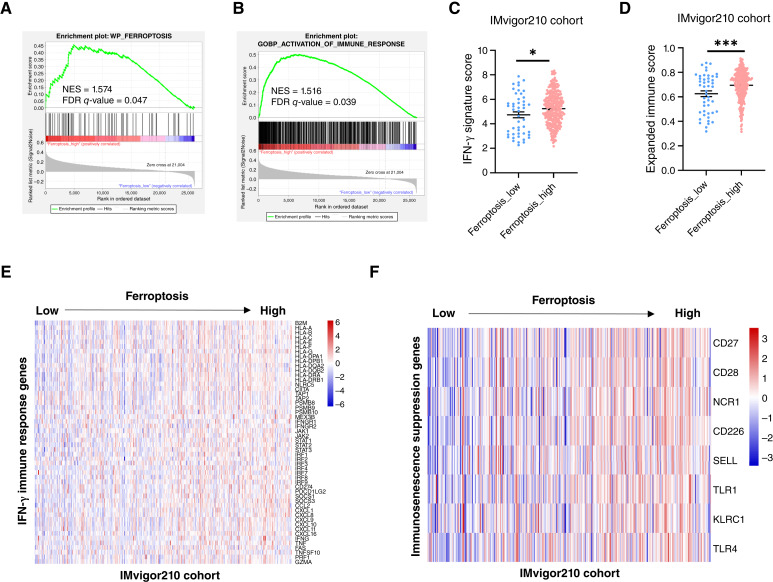
Ferroptosis-high tumors showed attenuated immunosenescence and enhanced immune response. **A** and **B,** Gene set enrichment analysis to examine ferroptosis enrichment and activation of the immune response in ferroptosis-high and ferroptosis-low tumors in the IMvigor210 cohort. **C** and **D,** Comparison of the IFN-γ signature score and expanded immune score of ferroptosis-low and ferroptosis-high tumors in the IMvigor210 cohort. **E,** Heatmap shows the correlation between ferroptosis and the expression of IFN-γ immune response signature genes in tumors of the IMvigor210 cohort. **F,** Heatmap shows the correlation between ferroptosis and the expression of immunosenescence suppressor genes in tumors of the IMvigor210 cohort. *, *P* < 0.05; ***, *P* < 0.001. NES, normalized enrichment scale.

To investigate the mechanism underlying the improved immunotherapy outcomes associated with ferroptosis, we first confirmed that the ferroptosis gene set was significantly enriched in ferroptosis-high tumors (Fig. 3A). Gene set enrichment analysis further revealed that pathways involved in immune activation were also enriched in ferroptosis-high tumors (Fig. 3B). Previous studies have demonstrated that the IFN-γ signature is predictive of anti–PD-1 therapy efficacy (45). In the IMvigor210 cohort of patients with urothelial cancer, we observed that ferroptosis-high tumors exhibited significantly elevated IFN-γ signature scores and expanded immune scores compared with ferroptosis-low tumors (Fig. 3C and D). A prior study identified a panel of IFN-γ responsive genes that are upregulated following IFN-γ treatment (46). We assessed the expression of these genes and found them to be markedly elevated in ferroptosis-high tumors, supporting the notion of an enhanced immune response (Fig. 3E). Moreover, analysis of gene expression revealed that ferroptosis-high tumors upregulated markers linked to the suppression of immunosenescence, supporting a role for ferroptosis in mitigating immunosenescence (Fig. 3F). Collectively, these findings indicate that ferroptosis promotes antitumor immunity by enhancing IFN-γ–mediated immune activation and suppressing immunosenescence within the tumor microenvironment.

### Ferroptosis is associated with increased immunogenicity and favorable tumor immune microenvironment subtypes

**Figure 4:**
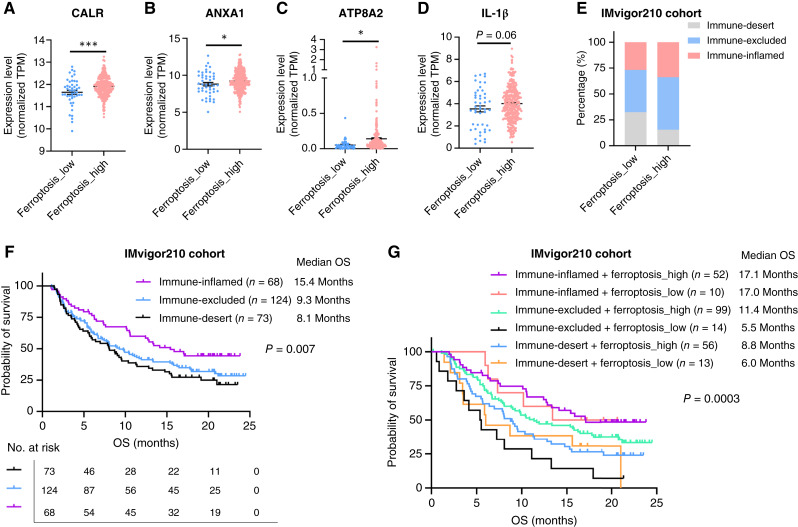
Ferroptosis is associated with increased immunogenicity and favorable TIME subtypes. **A–D,** Compare the expression of ICD biomarker genes in ferroptosis-low and ferroptosis-high tumors in the IMvigor210 cohort. **E,** Examine the proportions of TIME subtypes in ferroptosis-low and ferroptosis-high tumors in the IMvigor210 cohort. **F,** OS analysis of patients based on TIME subtypes in the IMvigor210 cohort. **G,** OS analysis of patients based on cancer-immune phenotypes and ferroptosis levels in tumor tissue in the IMvigor210 cohort. *, *P* < 0.05; ***, *P* < 0.001. ANXA1, annexin A1; ATP8A2, ATPase phospholipid transporting 8A2; CALR, calreticulin; TPM, transcripts per million.

ICD enhances the immunogenicity of tumor cells and can promote antitumor immune responses. Several molecular markers of ICD, such as calreticulin, annexin A1, ATPase phospholipid transporting 8A2, and IL-1β, have been identified. We examined the expression of these markers in the IMvigor210 cohort and found that ferroptosis-high tumors showed significantly elevated expression of ICD biomarkers, suggesting enhanced immunogenicity compared with ferroptosis-low tumors (Fig. 4A–D). Tumor immune microenvironment (TIME) subtypes (immune-inflamed, immune-excluded, and immune-desert) have been proposed to delineate the mechanisms of immune evasion (52). Among these, immune-inflamed tumors are generally more responsive to immunotherapy (53). Previous studies have shown that ICD can convert immune-excluded tumors into immune-inflamed tumors by increasing tumor immunogenicity (54). We, therefore, analyzed the distribution of TIME subtypes in the IMvigor210 cohort and found that ferroptosis-high tumors had a higher proportion of immune-inflamed and immune-excluded tumors compared with ferroptosis-low tumors (Fig. 4E). Survival analysis confirmed that patients with immune-inflamed tumors had the most favorable prognosis following immunotherapy, whereas those with immune-desert tumors had the worst prognosis (median OS: 15.4 vs. 9.3 vs. 8.1 months; *n* = 265; *P* = 0.0007; Fig. 4F). Importantly, when the ferroptosis activity model was integrated with TIME subtypes, we found that although it did not further stratify OS in patients with immune-inflamed tumors—who already exhibited favorable outcomes—it significantly refined prognostic predictions in patients with immune-excluded or immune-desert tumors. Notably, ferroptosis-high tumors within these subtypes showed improved OS compared with ferroptosis-low counterparts (median OS: 11.4 vs. 5.5; 8.8 vs. 6.6 months; *n* = 182; *P* = 0.0003; Fig. 4G). Collectively, these findings demonstrate that ferroptosis is associated with increased immunogenicity and enhanced immune infiltration, which together contribute to improved responses to immunotherapy.

## Discussion

Precision oncology has fundamentally reshaped cancer treatment paradigms (55, 56). Although chemotherapy continues to serve as the primary treatment for many patients, advances in genomic profiling have expanded the use of targeted therapies and immunotherapies to a growing subset of patients (5, 57, 58). Characterizing the molecular profiles of tumor tissues may provide novel therapeutic targets for cancers (59–61). In this study, we established a model based on the expression of the downstream targets of ferroptosis in tumor tissue, enabling the stratification of patients into ferroptosis-high and ferroptosis-low groups. We validated the prognostic significance of ferroptosis in eight independent cohorts of patients with cancer receiving immunotherapy. Furthermore, we demonstrated that ferroptosis enhances the antitumor immune response by increasing tumor immunogenicity and suppressing immunosenescence. To our knowledge, this study is the first to demonstrate the predictive value of ferroptosis in determining treatment outcomes in large cohorts of patients receiving immunotherapy.

Recent studies have shown that effective immunotherapy can induce peroxidation and ferroptosis in tumor cells, thereby enhancing therapeutic efficacy (24, 62). Moreover, the administration of early ferroptotic tumor cells has been shown to promote tumor regression by reprogramming the TIME (25). Meanwhile, blockage of the tyrosine-protein kinase receptor TYRO3 induced ferroptosis in tumor cells and increased sensitivity to immunotherapy (63). Furthermore, redox lipid reprogramming increased the vulnerability of tumor-associated macrophages to ferroptosis, underscoring the potential of targeting stromal cells within the tumor microenvironment to enhance antitumor immunity (64). In this study, we uncovered an unanticipated role of ferroptosis as a predictor of response to immunotherapy. Although immunotherapy has demonstrated promising efficacy in patients with melanoma with brain metastases, its benefit remains limited in those with liver metastases (50, 65). Consistently, our analysis revealed that patients with liver metastases exhibited poorer survival outcomes compared with those with other metastases. The reduced efficacy is partially attributed to the elimination of T cells by specific macrophage populations within liver metastases, contributing to the development of a systemic immune desert phenotype (50). We further stratified patients with nonhepatic metastases into ferroptosis-low and ferroptosis-high groups in both urothelial cancer and melanoma cohorts. Among this subset, only patients with high ferroptosis exhibited favorable prognoses. In contrast, those with low ferroptosis had poor outcomes despite the absence of liver metastases. This finding underscores the translational potential of our study as it identifies a subset of patients, such as those with low TMB, who might otherwise be deemed nonresponsive to immunotherapy. Tumor microenvironment plays pivotal roles in regulating tumor progression, cachexia, and drug resistance (66, 67). Further studies may investigate whether and how ferroptosis promotes cancer progression via reprogramming the microenvironment.

The causal relationship between ferroptosis and antitumor immunity warrants further study. Ferroptosis may act as both a driver and a consequence of enhanced antitumor immune surveillance. Nevertheless, from a clinical perspective, the predictive value of ferroptosis remains relevant regardless of whether it is a cause or a consequence of enhanced antitumor immunity. Preclinical studies have demonstrated that activated CD8^+^ T cells can induce ferroptosis in tumor cells (24). In patients with cancer, reduced expression of SLC3A2/SLC7A11 in tumor tissues is associated with a “T-cell inflamed” phenotype (24). These findings suggest a positive feedforward circuit between tumor cells and cytotoxic T cells, which is partially regulated by ferroptosis. Specifically, the induction of ferroptosis in tumor cells promotes T-cell infiltration and activity, which in turn enhances ferroptosis in tumor cells. Importantly, unlike other untargetable biomarkers such as TMB and deficient mismatch repair, ferroptosis is pharmacologically targetable. Preclinical studies have demonstrated synergistic effects when ferroptosis inducers are combined with other treatment modalities, including radiotherapy and chemotherapy (68, 69). The current study provides additional rationale for combining ferroptosis inducers with immunotherapy in the treatment of cancers.

The method we used to construct the ferroptosis model is straightforward and clinically accessible. We normalized the expression of each ferroptosis-related gene across all samples and then summed these values to generate a composite score. This simple yet effective approach makes the model well suited for clinical implementation and underscores its strong translational potential. Notably, although we acknowledge that employing a universal cutoff value would enhance convenience for prospective clinical application, this study used different thresholds to determine high/low in different cohorts. Indeed, a universal threshold would be particularly appropriate in scenarios in which the biomarker signature is applied to cohorts of patients with the same cancer type who have received similar treatment regimens. However, in our study, the included patient cohorts vary in cancer types and treatment modalities. For example, although all patients underwent immunotherapy, treatment approaches included anti–PD-1 therapy, anti-CTLA4 therapy, and T-cell infusion therapy. Therefore, we believe that customized cutoff values are more suitable to accurately capture cohort-specific biological and clinical variability.

Our study has several limitations. We analyzed transcriptomic alterations at the bulk tumor tissue level, which encompasses heterogeneous cell populations beyond tumor cells. This approach was necessary because, currently, few clinical datasets provide both single-cell RNA-seq data and matched immunotherapy outcomes. Some studies offer single-cell RNA-seq data without clinical outcome information (70), whereas others focus exclusively on immune cells, omitting tumor cells entirely (71). Future studies leveraging single-cell resolution may offer more precise insights into the role of ferroptosis in cancer immunotherapy. Additionally, further investigation is warranted to determine whether ferroptosis agonists can enhance clinical responses to immunotherapy.

In summary, patients with ferroptosis-high tumors exhibited superior treatment outcomes compared with those with ferroptosis-low tumors following immunotherapy. This study offers novel insights into the pivotal role of ferroptosis in enhancing the effectiveness of cancer immunotherapy. Our findings support the therapeutic rationale for inducing tumor ferroptosis as a strategy to improve the efficacy of immunotherapy.

## Supplementary Material

Supplementary Fig. S1 Supplementary Fig. S1. Correlation between ferroptosis level and overall survival of patients in Van-Allen cohort and Hugo cohort.

Supplementary Fig. S2 Supplementary Fig. S2. Progression-Free Survival of patients in Van-Allen cohort and Lauss cohort based on ferroptosis level.

Supplementary Fig. S3 Supplementary Fig. S3. Correlation between ferroptosis score and overall survival in cancer patients without receiving immunotherapy.

Supplementary Fig. S4 Supplementary Fig. S4. The association between tumor ferroptosis level and immunotherapy response in gastric cancer.

Supplementary Fig. S5 Supplementary Fig. S5. Prognosis and TMB of non-small cell lung cancer (NSCLC) are associated with ferroptosis level.

Supplementary Fig. S6 Supplementary Fig. S6. Correlation between TMB and ferroptosis in patients received immunotherapy.

Supplementary Table S1 Supplementary Table S1
