# Supplementary material for: Evaluation of Ferroptosis as a Biomarker to Predict Treatment Outcomes of Cancer Immunotherapy
Source: Cancer Res Commun. 2025 Aug 6;5(8):1288–97. doi: 10.1158/2767-9764.CRC-25-0268 (PMC12326525; doi:10.1158/2767-9764.CRC-25-0268)
Supplement: Supplementary Fig. S1 — Correlation between ferroptosis level and overall survival of patients in Van-Allen cohort and Hugo cohort. [file crc-25-0268_supplementary_fig.s1_suppsf1.pdf]

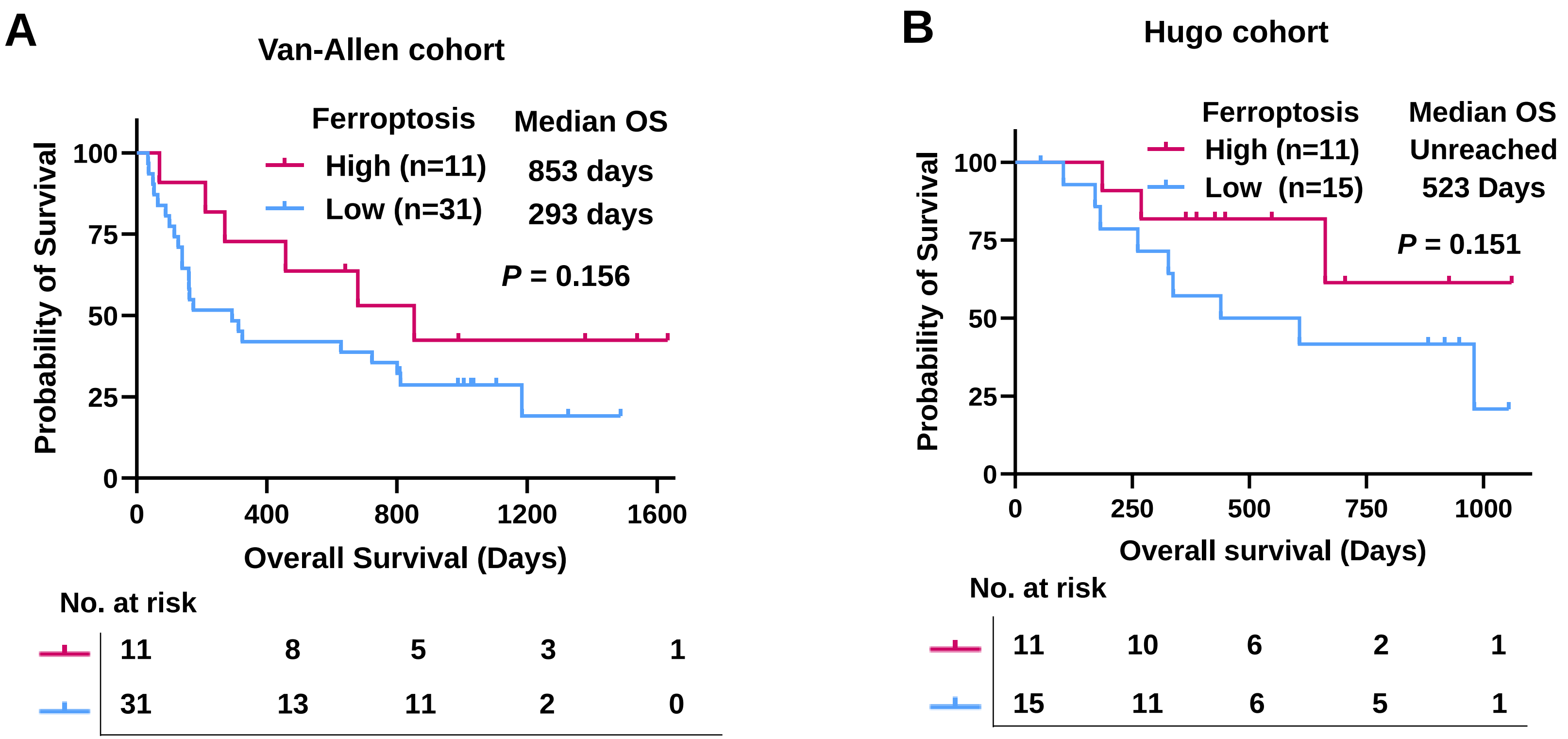

Supplementary Fig. S1. Correlation between ferroptosis level and overall survival of patients in Van-Allen cohort and Hugo cohort. A, Overall survival analysis of patients based on ferroptosis level in tumor tissue in Van-Allen cohort (melanoma). **B**, Overall survival analysis of patients based on ferroptosis level in tumor tissue in Hugo cohort (melanoma).
