# Supplementary material for: Evaluation of Ferroptosis as a Biomarker to Predict Treatment Outcomes of Cancer Immunotherapy
Source: Cancer Res Commun. 2025 Aug 6;5(8):1288–97. doi: 10.1158/2767-9764.CRC-25-0268 (PMC12326525; doi:10.1158/2767-9764.CRC-25-0268)
Supplement: Supplementary Fig. S2 — Progression-Free Survival of patients in Van-Allen cohort and Lauss cohort based on ferroptosis level. [file crc-25-0268_supplementary_fig.s2_suppsf2.pdf]

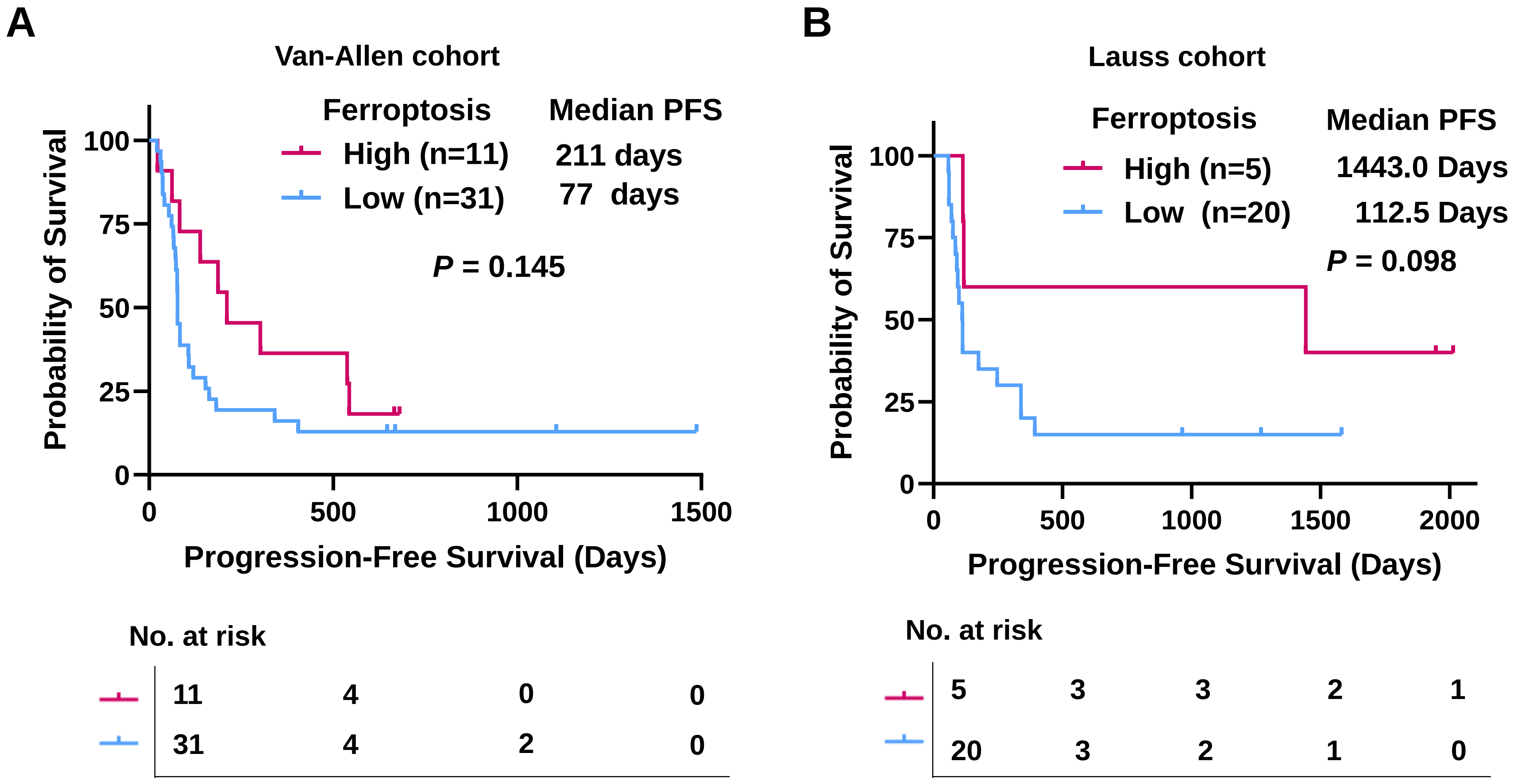

Supplementary Fig. S2. Progression-Free Survival of patients in Van-Allen cohort and Lauss cohort based on ferroptosis level. A, Progression-Free Survival of patients in the Van-Allen cohort based on ferroptosis level. **B**, Progression-Free Survival of patients in the Lauss cohort based on ferroptosis level. Log-rank test was applied for the survival analysis.
