# Supplementary material for: Evaluation of Ferroptosis as a Biomarker to Predict Treatment Outcomes of Cancer Immunotherapy
Source: Cancer Res Commun. 2025 Aug 6;5(8):1288–97. doi: 10.1158/2767-9764.CRC-25-0268 (PMC12326525; doi:10.1158/2767-9764.CRC-25-0268)
Supplement: Supplementary Fig. S3 — Correlation between ferroptosis score and overall survival in cancer patients without receiving immunotherapy. [file crc-25-0268_supplementary_fig.s3_suppsf3.pdf]

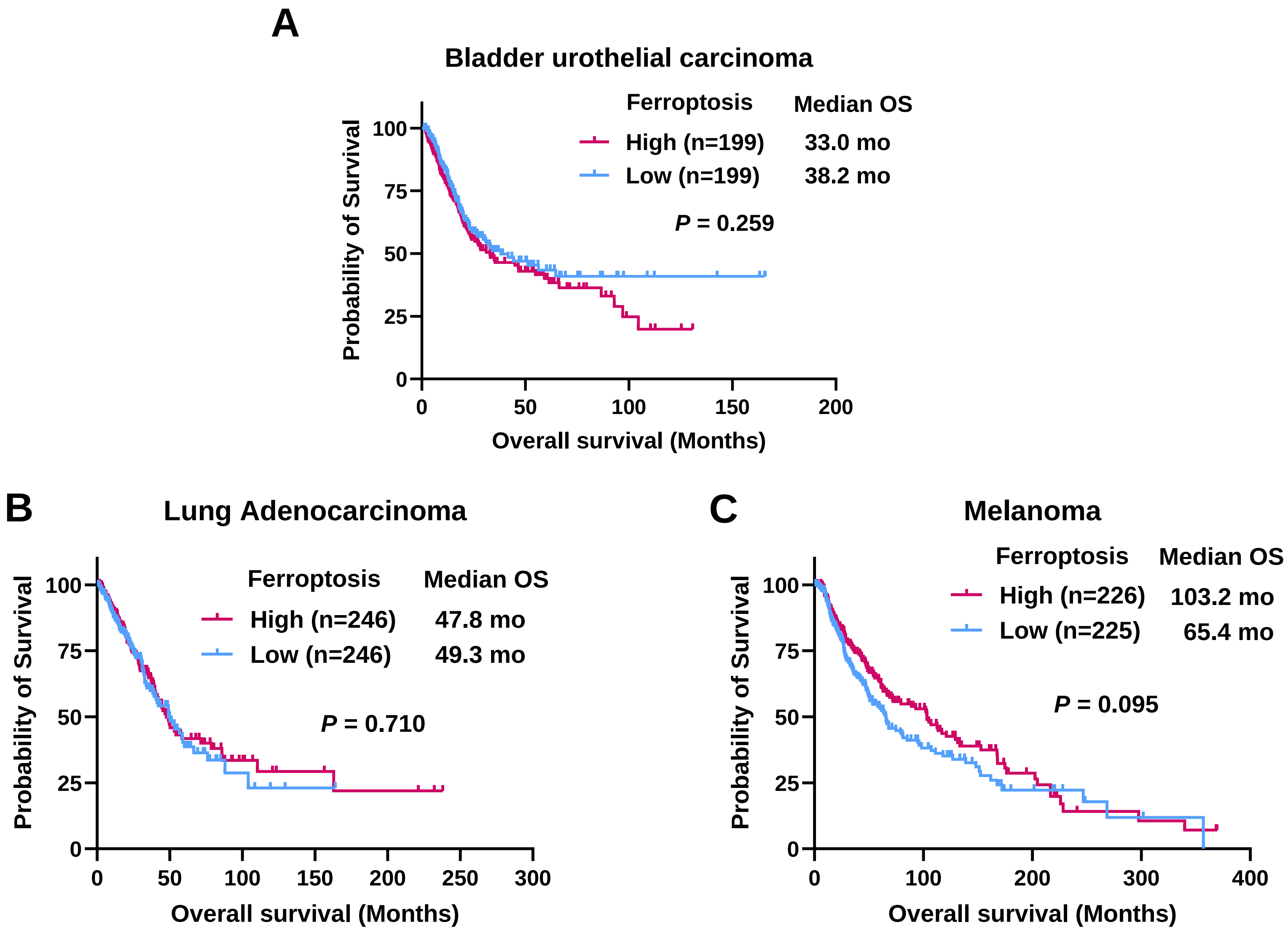

Supplementary Fig. S3. Correlation between ferroptosis score and overall survival in cancer patients without receiving immunotherapy. Overall survival analysis based on ferroptosis score in patients with (A) bladder cancer, (B) lung cancer, and (C) melanoma receiving standard-of-care (non-immunotherapy) treatment.
