# Supplementary material for: Evaluation of Ferroptosis as a Biomarker to Predict Treatment Outcomes of Cancer Immunotherapy
Source: Cancer Res Commun. 2025 Aug 6;5(8):1288–97. doi: 10.1158/2767-9764.CRC-25-0268 (PMC12326525; doi:10.1158/2767-9764.CRC-25-0268)
Supplement: Supplementary Fig. S4 — The association between tumor ferroptosis level and immunotherapy response in gastric cancer. [file crc-25-0268_supplementary_fig.s4_suppsf4.pdf]

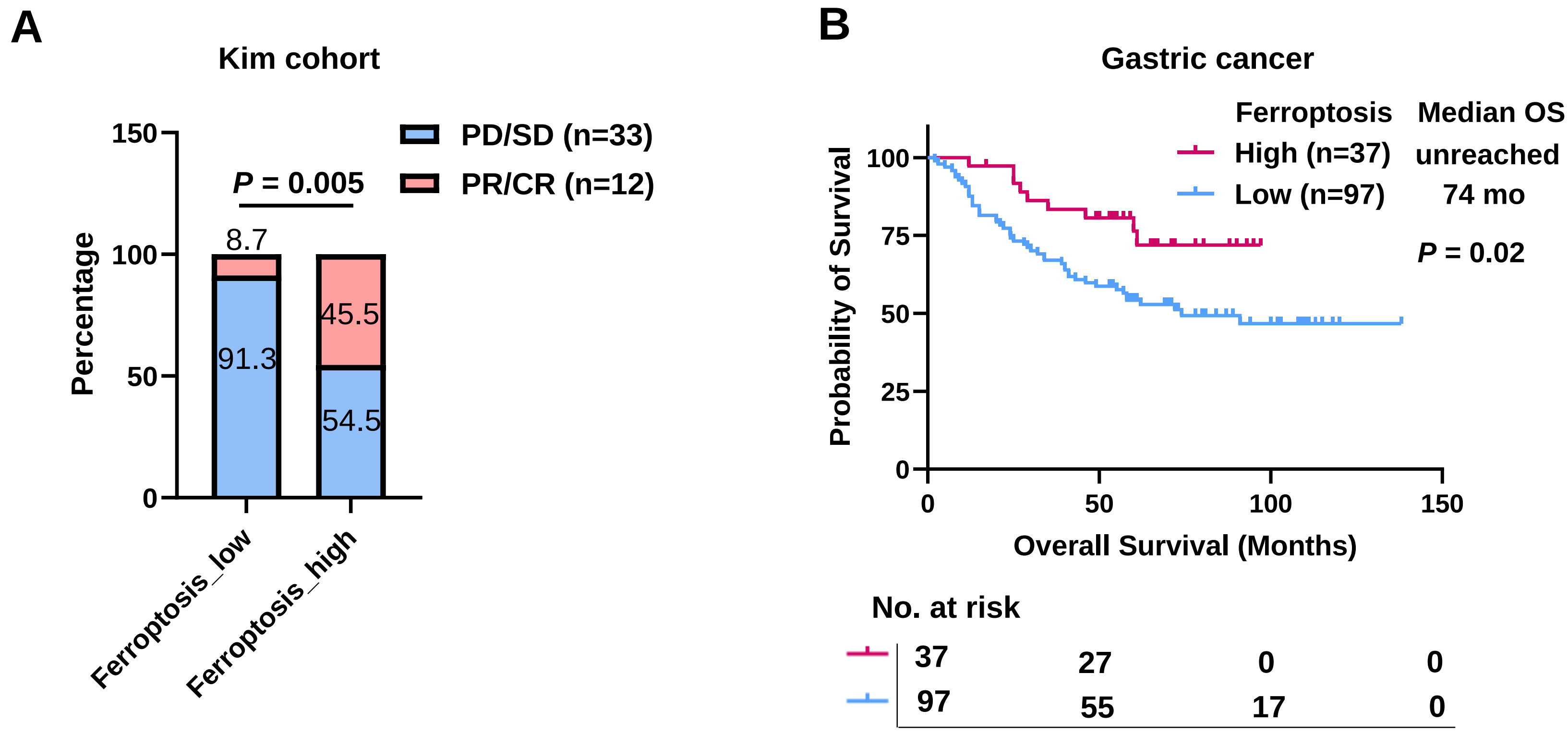

Supplementary Fig. S4. The association between tumor ferroptosis level and immunotherapy response in gastric cancer. A, Treatment response of immunotherapy in Kim cohort based on the level of ferroptosis in tumor tissue. **B**, Overall survival analysis of patients receiving chemotherapy for gastric cancer. Log-rank test was applied for the survival analysis.
