# Supplementary material for: Evaluation of Ferroptosis as a Biomarker to Predict Treatment Outcomes of Cancer Immunotherapy
Source: Cancer Res Commun. 2025 Aug 6;5(8):1288–97. doi: 10.1158/2767-9764.CRC-25-0268 (PMC12326525; doi:10.1158/2767-9764.CRC-25-0268)
Supplement: Supplementary Fig. S5 — Prognosis and TMB of non-small cell lung cancer (NSCLC) are associated with ferroptosis level. [file crc-25-0268_supplementary_fig.s5_suppsf5.pdf]

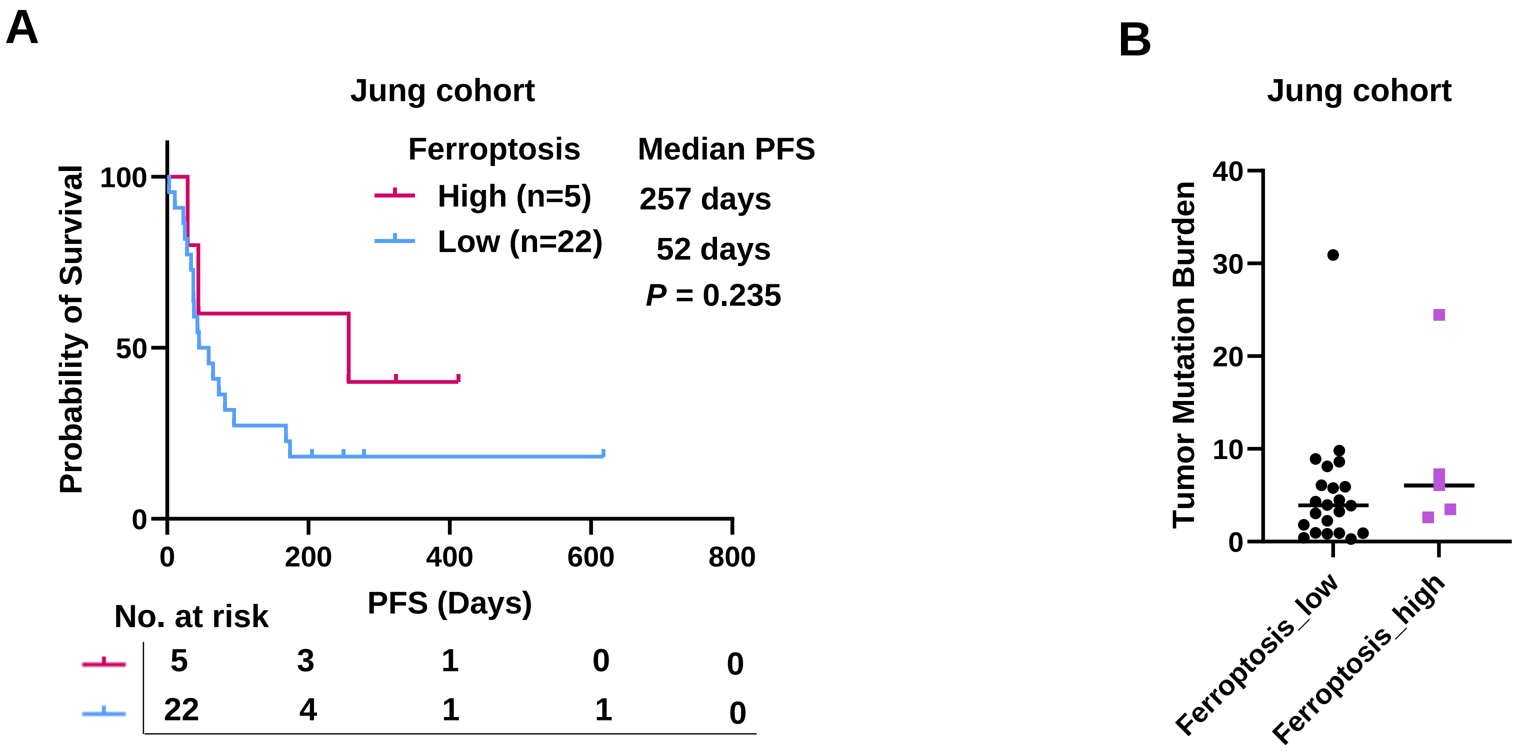

Supplementary Fig. S5. Prognosis and TMB of non-small cell lung cancer (NSCLC) are associated with ferroptosis level. A, Progression-free survival of patients in Jung cohort based on ferroptosis level. **B**, Association between TMB and ferroptosis in Jung cohort. Log-rank test was applied for the survival analysis.
