# Supplementary material for: Evaluation of Ferroptosis as a Biomarker to Predict Treatment Outcomes of Cancer Immunotherapy
Source: Cancer Res Commun. 2025 Aug 6;5(8):1288–97. doi: 10.1158/2767-9764.CRC-25-0268 (PMC12326525; doi:10.1158/2767-9764.CRC-25-0268)
Supplement: Supplementary Fig. S6 — Correlation between TMB and ferroptosis in patients received immunotherapy. [file crc-25-0268_supplementary_fig.s6_suppsf6.pdf]

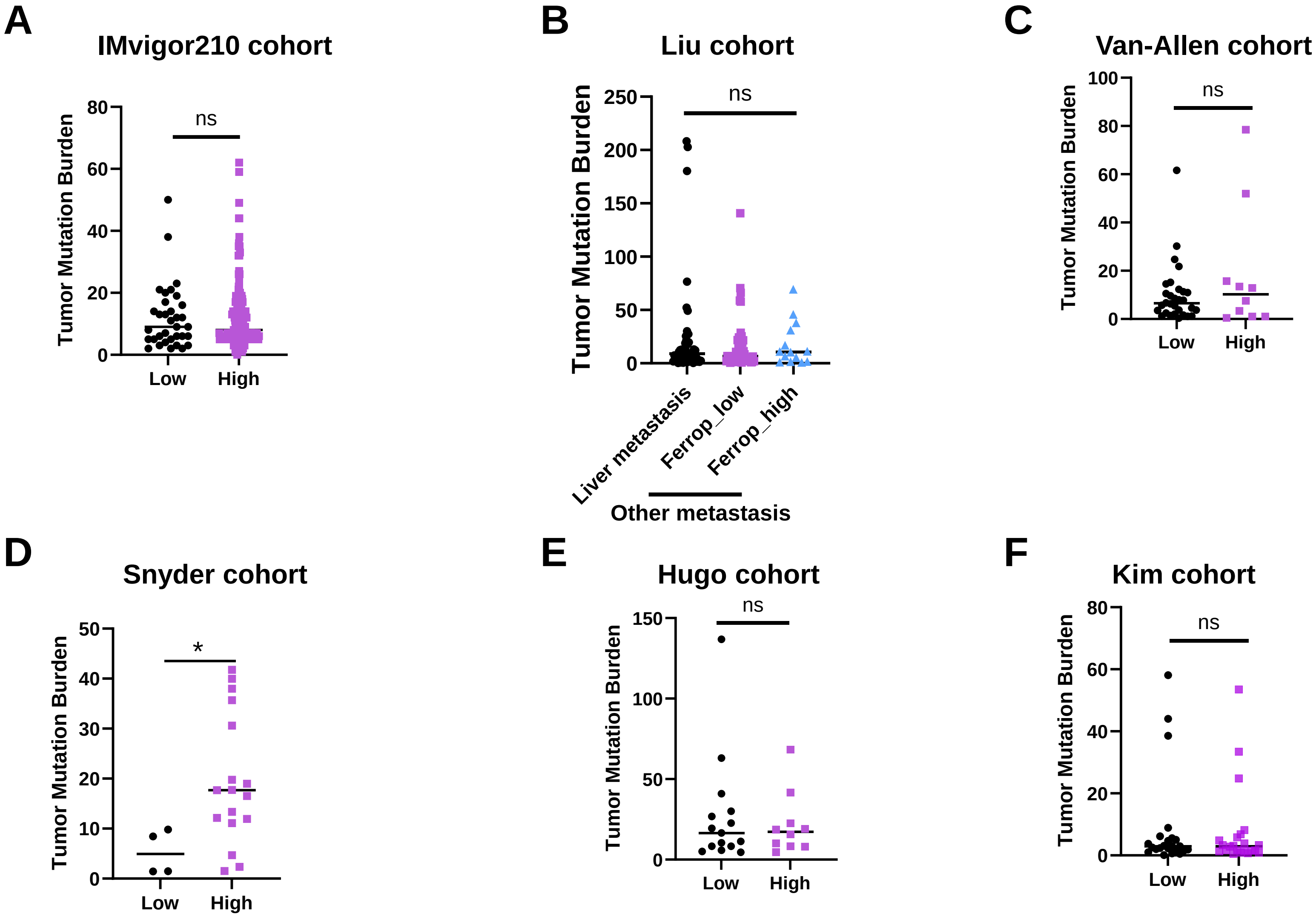

Supplementary Fig. S6. Correlation between TMB and ferroptosis in patients received immunotherapy. A, Correlation between TMB and ferroptosis in IMvigor210 cohort. **B**, Correlation between TMB and ferroptosis in Liu cohort. **C**, Correlation between TMB and ferroptosis in Van-Allen cohort. **D**, Correlation between TMB and ferroptosis in Snyder cohort. **E**, Correlation between TMB and ferroptosis in Hugo cohort. **F**, Correlation between TMB and ferroptosis in Kim cohort.
