# Supplementary material for: Evaluation of Ferroptosis as a Biomarker to Predict Treatment Outcomes of Cancer Immunotherapy
Source: Cancer Res Commun. 2025 Aug 6;5(8):1288–97. doi: 10.1158/2767-9764.CRC-25-0268 (PMC12326525; doi:10.1158/2767-9764.CRC-25-0268)
Supplement: Supplementary Table S1 [file crc-25-0268_supplementary_table_s1_suppst1.docx]

**Supplementary Table S1. Information of the cohorts included in this study.**

| Cohort | Number of cases | Cancer types | Therapy type | Accession ID |
| --- | --- | --- | --- | --- |
| IMvigor210 | 348 | Urothelial cancers | Anti-PD1 | PMID: 29443960 |
| Snyder cohort | 21 | Melanoma | Anti-CTLA4 | PMID: 25409260 |
| Liu cohort | 121 | Melanoma | Anti-PD1 | PMID: 31792460 |
| Lauss cohort | 25 | Melanoma | T cell infusion | GSE100797 |
| Van-Allen cohort | 42 | Melanoma | Anti-CTLA4 | PMID: 26359337 |
| Hugo cohort | 26 | Melanoma | Anti-PD1 | GSE78220 |
| Kim cohort | 45 | Gastric cancer | Anti-PD1 | PRJEB25780 |
| Jung cohort | 27 | Lung cancer | Anti-PD1 | GSE135222 |
